# Ni-Doped Pr_0.5_Ba_0.5_CoO_3+δ_ Perovskite with Low Polarization Resistance and Thermal Expansivity as a Cathode Material for Solid Oxide Fuel Cells

**DOI:** 10.3390/molecules30071482

**Published:** 2025-03-27

**Authors:** Runze Sun, Songbo Li, Lele Gao, Shengli An, Zhen Yan, Huihui Cao, Qiming Guo, Mengxin Li

**Affiliations:** 1 School of Chemistry and Chemical Engineering, Inner Mongolia University of Science and Technology, Baotou 014010, China; 18264331178@163.com (R.S.); m13964589510@163.com (Q.G.); 15175447968@163.com (M.L.); 2 Rare Earth Advanced Materials Technology Innovation Center, Inner Mongolia Northern Rare Earth Advanced Materials Technology Innovation Co., Ltd., Baotou 014030, China; gaolele@reamtic.cn (L.G.); yanzhen@reamtic.cn (Z.Y.); caohuihui@reamtic.cn (H.C.); 3 School of Rare Earth Industry, Inner Mongolia University of Science and Technology, Baotou 014010, China; san@imust.edu.cn

**Keywords:** solid oxide fuel cell, Ni doping, cathode material, electrochemistry

## Abstract

Solid oxide fuel cells (SOFCs) have become promising devices for converting chemical energy into electrical energy. Altering the microstructure of cathode materials to enhance the activity and stability of the oxygen reduction reaction is particularly important. Herein, Pr_0.5_Ba_0.5_Co_1−X_Ni_X_O_3+δ_ with a tetragonal perovskite structure was synthesized through the sol–gel method. The polarization resistance of the symmetrical half-cell with Pr_0.5_Ba_0.5_Co_0.9_Ni_0.1_O_3+δ_ as the cathode was 0.041 Ω·cm^2^ at 800 °C and 0.118 Ω·cm^2^ lower than that of the symmetrical cell with Pr_0.5_Ba_0.5_CoO_3+δ_ as the cathode, indicating that the Pr_0.5_Ba_0.5_Co_1−X_Ni_X_O_3+δ_ cathode material had high catalytic activity during the electrochemical reaction. The results of electron paramagnetic resonance revealed that the concentration of oxygen vacancies increased as the Ni doping amount increased to 0.15. As a result of the increase in the Ni doping amount, the thermal expansion coefficient of the Pr_0.5_Ba_0.5_CoO_3+δ_ cathode material was effectively reduced, resulting in improved matching between the cathode and electrolyte material. The power density of the single cell increased by 69 mW·cm^−2^. Therefore, Pr_0.5_Ba_0.5_Co_1−X_Ni_X_O_3+δ_ is a promising candidate cathode material for high-performance SOFCs.

## 1. Introduction

Solid oxide fuel cells (SOFCs) can directly convert chemical energy into electrical energy and are promising energy conversion devices [1]. A single SOFC consists of an electrolyte, a cathode, and an anode. Among various factors, the polarization resistance of the cathode, which is the energy required to overcome the oxygen reduction reaction (ORR), has the greatest effect on reaction activity. Therefore, reducing cathode polarization resistance and improving cathode performance are effective ways to increase the power generation efficiency of SOFCs [2]. Exploring new cathode materials with high catalytic activity is a research hotspot in the field of SOFCs.

Given their excellent electrochemical properties and mixed ion–electron conductivity, perovskite oxides are attracting considerable attention as a new type of mixed ion–electron conductor (MIEC) [3]. Extending the ORR region to the entire electrode surface beyond the electrolyte–electrode–gas three-phase boundary is beneficial to improve electrode performance and has high research value in the field of SOFC cathode materials. Woo et al. [4] found that the conductivity of SOFC cathode materials with Pr and La at the A site is higher than that of compounds with Sm and Gd and the conductivity of Co-based compounds at the B site is considerably higher than that of Fe-based materials [5]. PBC is widely used as an air electrode material because of its high oxygen diffusion coefficient and excellent chemical reaction kinetics [6]. Gu et al. [7] showed that the polarization impedance of PrBaCo_2_O_5+δ_ cathode materials at 600 °C was only 0.07 Ω·cm^2^. Co-based materials have become a hot topic in the research on SOFC cathode materials due to their excellent electrical conductivity and power density [8]. However, given their high thermal expansion coefficient, they have poor thermal matching with electrolyte materials [9]. Bai et al. [10] revealed that the thermal expansion coefficient (TEC) of the cathode material reduced after adding 0.5 Fe at the B position in PrBa_0.5_Sr_0.5_Co_1.5_Fe_0.5_O_5+δ_, with better thermal matching with the electrolyte, and the electrochemical performance was improved, which increased the service life of the cell. Doping Ta [11], Ni [12], Nb [13], Cu [14], and Fe [10] at the B site of Co-based materials can promote the ORR at the electrode and improve electrochemical performance and catalytic activity. Ni doping at the B site can reduce proton migration capacity, increase oxygen vacancy concentration, and improve proton absorption and ORR catalytic activity. Zhu et al. [12] demonstrated that the Pr_0.7_Ba_0.3_Co_0.6_Fe_0.2_Ni_0.2_O_3+δ_ material has good ORR activity in dry and wet air.

In this study, PrBaCo_1−X_Ni_X_O_3+δ_ (PBCNi_x_, X = 0, 0.05, 0.1, 0.15) cathode materials were prepared through the sol–gel method, with Ni as the doping element. The synthesized perovskite PBCNi_X_ cathode material was characterized by using X-ray diffraction (XRD), X-ray photoelectron spectroscopy (XPS), scanning electron microscopy (SEM), transmission electron microscopy (TEM), and energy disperse spectroscopy (EDS). The effects of Ni content on the microstructure and electrochemical properties of the PBC cathode materials were investigated.

## 2. Results and Discussion

**Figure 1:**
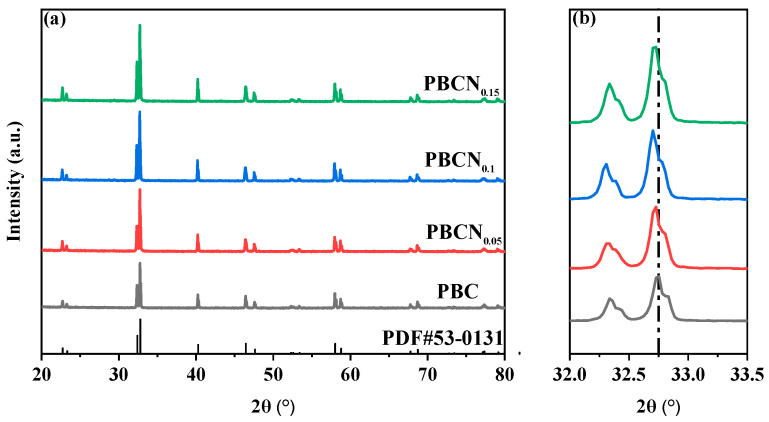
(**a**) XRD patterns of cathode materials PBCNi_X_. (**b**) XRD local magnification image.

**Figure 2:**
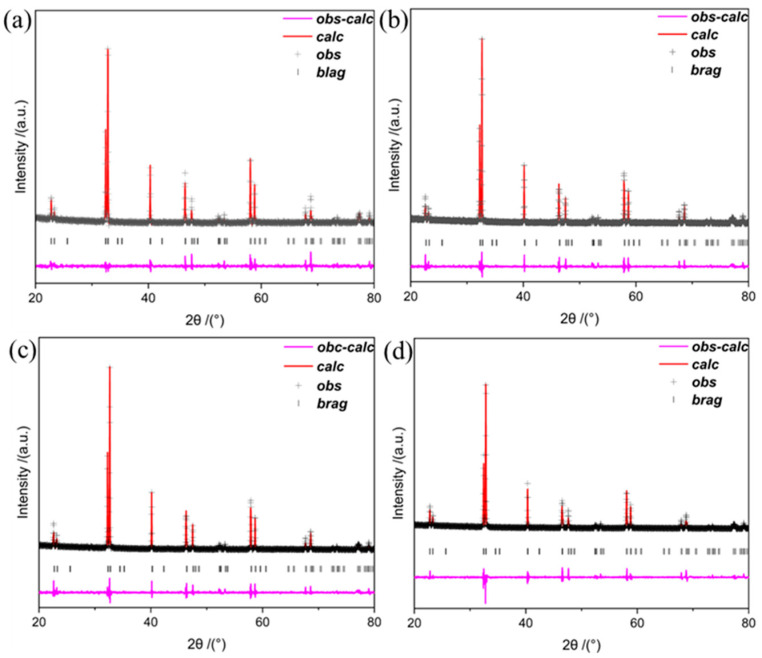
Rietveld XRD refined patterns: (**a**) PBC; (**b**) PBCNi_0.05_; (**c**) PBCNi_0.1_; (**d**) PBCNi_0.15_.

The XRD pattern of PBCNi_X_ (X = 0, 0.05, 0.1, 0.15) is provided in Figure 1a. The diffraction peaks were narrow and sharp, and the main diffraction peak position of the Ni-doped sample was consistent with the structure of Pr_0.5_Ba_0.5_CoO_3+δ_ standard card (PBC, PDF#53-0131), showing a typical quartet-phase perovskite structure [15]. These results demonstrated that the perovskite PBCNi_X_ materials were prepared successfully and that no secondary phase had formed. Figure 1b presents an enlarged cross-section of 2θ = 32–33.5°. The characteristic peaks of the PBCNi_X_ materials shifted to a low angle with the doping of Ni. Given that the radius of the Ni^2+^ ion was greater than that of the Co^3+^ ion, the XRD diffraction peak caused by lattice expansion shifted to a low angle, and the deviation of the diffraction peak gradually increased with the increase in the doping amount. The Rietveld method was used to refine the XRD pattern of PBC to study the effect of Ni ion doping on the crystal structure of PBC further. Figure 2 shows the Rietveld-refined pattern of PBCNi_X_, which was consistent with the XRD patterns. Table 1 shows that materials with high Ni doping amounts had large cell volumes: PBC, PBCNi_0.05_, PBCNi_0.1_, and PBCNi_0.15_ had cell volumes of 116.237 Å, 116.722 Å, 117.002 Å, and 117.195 Å, respectively. The cell volume increased gradually because Ni^2+^ had a bigger radius than that of Co^3+^ and entered the cell to replace Co^3+^; thus, more oxygen vacancies could be formed. This indicated that the Ni ions successfully entered the lattice of the PBC cathode materials. The results showed that the prepared PBCNi_X_ had the same spatial structure as the undoped PBC, indicating that Ni doping did not change the original crystal structure, with a simple tetragonal structure being retained (P4/mmm).

**Figure 3:**
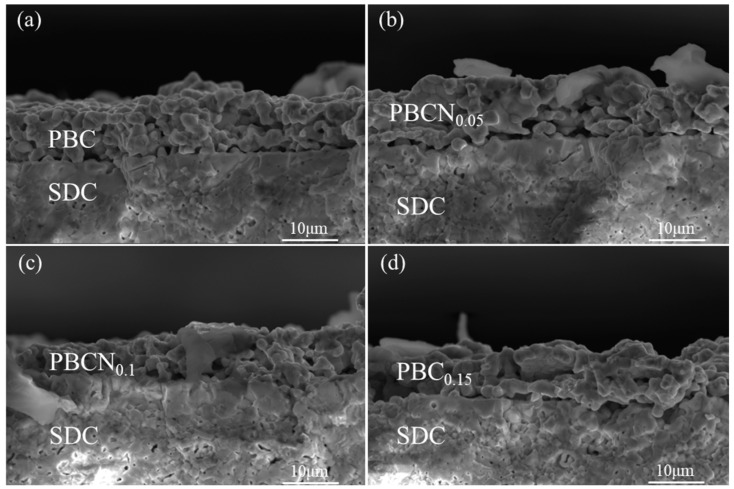
SEM images of symmetric cell with cathode material (**a**) PBC; (**b**) PBCNi_0.05_; (**c**) PBCNi_0.1_; (**d**) PBCNi_0.15_.

Figure 3 shows the SEM images of the symmetrical cells of PBC and the PBCNi_X_ cathode materials after calcination at 1100 °C and the subsequent electrochemical performance tests. The results indicated that Ni doping had no discernible effect on the structure of PBC. The prepared cathode materials were loose and porous and therefore had relatively high porosity. The formation of a porous structure in the cathode was conducive to providing additional active sites for ORR and offering supplemental gas diffusion channels, which were beneficial for gas exchange and diffusion. The adhesion between the cathode material and SDC electrolyte was good, and no delamination or fracture was observed, indicating that the cathode and SDC electrolyte had good thermal compatibility.

**Figure 4:**
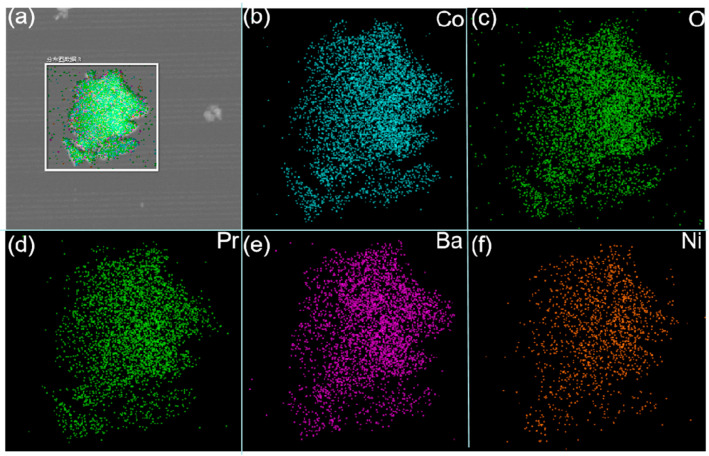
(**a**) EDS of PBCNi_0.1_ cathode surface. (**b**) Co element distribution. (**c**) O element distribution. (**d**) Pr element distribution. (**e**) Ba element distribution. (**f**) Ni element distribution.

Figure 4b–f show the element distribution maps of PBC and PBCNi_0.1_ as well as the corresponding energy dispersion spectra to illustrate the distribution of elements in the cathode materials doped with Ni ions. Table 2 shows the percentages of each element in the material. The results show that all elements in the synthesized materials were uniformly distributed, no element agglomeration occurred, the peaks of all elements were detectable, and the percentages of each element were in agreement with the stoichiometry. These findings further prove that Ni ions were effectively doped into the PBC materials.

**Figure 5:**
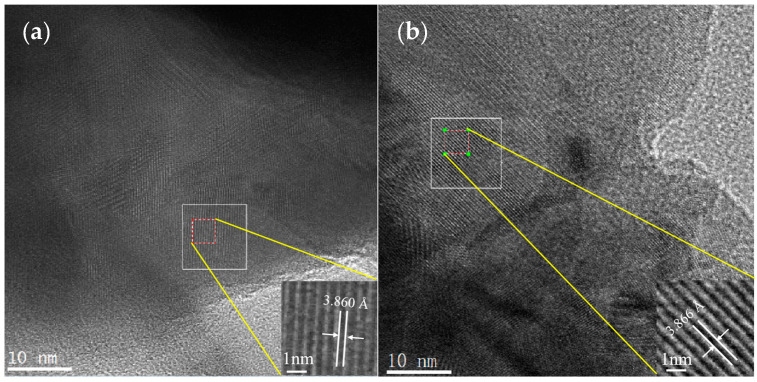
(**a**) TEM of PBC. (**b**) TEM of PBCNi_0.1_ cathode material.

Figure 5 shows the HR-TEM and locally amplified images. Figure 5a depicts that the diffraction fringe width of the substrate material (PBC) was 3.860 Å. Figure 5b illustrates that the diffraction fringe width of the cathode material with doping amount X = 0.1 was 3.866 Å. The TEM result was consistent with the refined XRD data. The synthesized PBCNi_0.1_ material was further shown to be centrosymmetric and had a simple tetragonal structure (P4/mmm).

**Figure 6:**
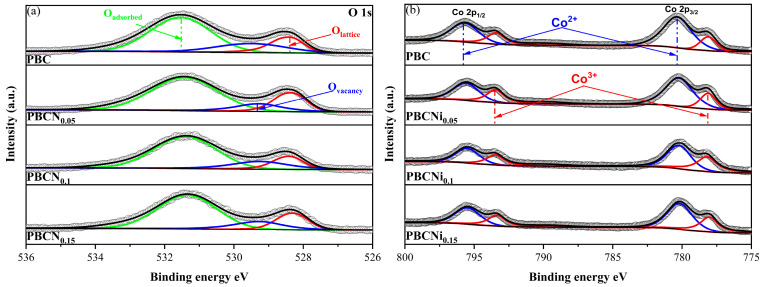
(**a**) XPS patterns of PBCNi_X_ O 1s orbit and (**b**) PBCNi_X_ Co 2p orbit (different colors mean different valences).

XPS was used for characterization to determine the surface composition of the PBCNi_X_ materials. The XPS spectral data of the O1s and Co2p orbitals in the PBCNi_X_ samples were fitted, and the corresponding spectra are provided in Figure 6. Figure 6a shows that the O element existed in three forms: lattice oxygen (*O_lattice_*, 528.54 eV), oxygen vacancy (*O_vacancy_*, 529.07 eV), and adsorbed oxygen (*O_adsorbed_*, 531.34 eV). According to the semi-quantitative analysis of XPS, the concentration of *O_vacancy_* increased from 8.88% to 11.36% (Table S1), which was the same as the EPR test result. This indicated that Ni doping increased the concentration of oxygen vacancies on the material surface. As shown in Figure 6b, low-valent Ni ions were doped into the PBC materials to replace Co. Co was induced to change from low valence to high valence, generating additional Co^3+^ (the content of Co^3+^ increased from 37.19% to 42.56%), as shown in Table S2, to maintain charge balance. Given that the ionic radius of the Ni^2+^ ions was larger than that of the Co^3+^ ions, additional oxygen vacancies were produced. This result was consistent with the findings of the previous analysis of the O1s orbital. Other peak fitting data are shown in Table S3.

The PBCNi_X_ cathode materials were tested through electron paramagnetic resonance (EPR), as illustrated in Figure S1. The oxygen vacancy signal increased with Ni doping, indicating that Ni doping increased the concentration of oxygen vacancies in the material and enhanced the catalytic activity of the oxygen [16]. In summary, Ni-doped samples could increase the oxygen vacancy content of the materials and are expected to become cathode materials with excellent electrical performance.

**Figure 7:**
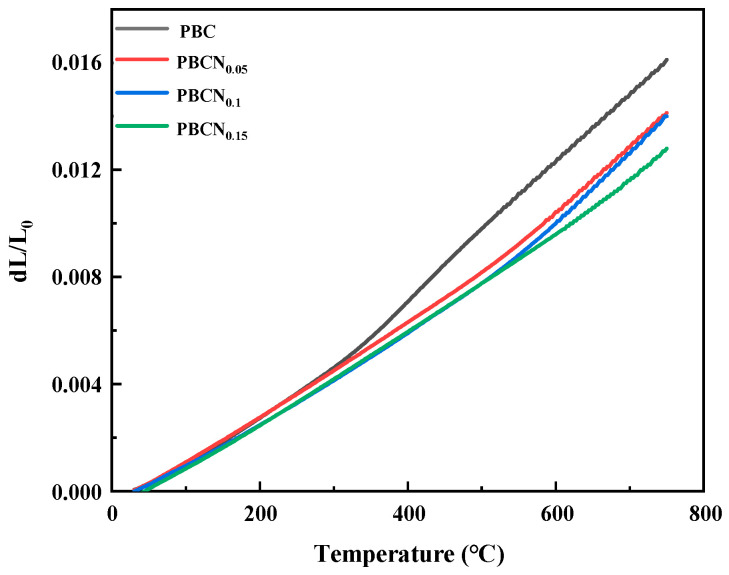
Thermal expansion diagram of PBCNi_X_ at 30–800 °C.

The influence of TEC on Co-based cathode materials is particularly important. It is necessary to give their TEC and electrolyte SDC better thermal matching to reduce the risk of cathode material fracture and increase the service life of the cell. Figure 7 shows that the average TEC values of the PBCNi_X_ (X = 0, 0.05, 0.1, 0.15) series of cathode materials within the temperature range of 30–800 °C were 22.0474 × 10^−6^ K^−1^ (X = 0), 19.598 × 10^−6^ K^−1^ (X = 0.05), 19.4837 × 10^−6^ K^−1^ (X = 0.1), and 18.0548 × 10^−6^ K^−1^ (X = 0.15). The TEC of SDC was 12.14 × 10^−6^ K^−1^ [17]. The doping of Ni reduced the average TEC of the PBC materials, making it close to that of SDC, indicating good thermal matching between the PBCNi_X_ cathode materials and SDC electrolyte. This thus minimized the risk of fracture caused by TEC mismatch between the electrolyte and cathode material and conferred the cell with good stability and a long service life.

**Figure 8:**
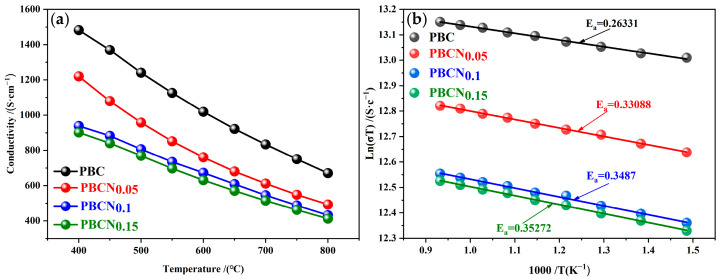
(**a**) The relationship between the conductivity of PBCNi_X_ and temperature. (**b**) Arrhenius curve of conductivity and temperature of PBCNi_X_.

Figure 8a shows the relationship between temperature and the electrical conductivity (σ) of the PBCNi_X_ materials in an air atmosphere within the temperature range of 400–800 °C. As the temperature increased, the conductivity of the sample decreased. The conductivity of the PBC and PBCNi_X_ series of cathode materials decreased in the range of 400–800 °C. At the same temperature, the electrical conductivity of the PBCNi_X_ series materials was lower than that of PBC because the electron transfer between Co^2+^ and Co^3+^ enhanced the electronic conductivity. With the increase in Ni doping content, the concentration of high-valent Co ions in the PBCNi_X_ cathode materials decreased, whereas that of oxygen vacancies increased, resulting in a decrease in the electrical conductivity of the materials. Figure 8b presents the Arrhenius plot of the electrical conductivity of the PBCNi_X_ cathode materials versus temperature within the temperature range of 400–800 °C. The activation energy *E_a_* of the PBCNi_X_ cathode materials with different Ni ion doping amounts was calculated by using the Arrhenius formula, as shown in Figure 8b [18].

(1) $$Ln\left(\sigma T\right)=LnA-\left(\frac{Ea}{kT}\right),$$

where *k* is the reaction rate constant at temperature *T*, which was 8.617 × 10^−5^; σ is the electrical conductivity; *A* is the pre-exponential factor; and *E_a_* is the conductivity activation energy. Consistent with the trend in the change in electrical conductivity, the conductivity activation energy of the PBCNi_X_ cathode materials continued to increase with the increase in the doping amount of Ni ions. Although electrical conductivity decreased due to the doping of Ni, it could still reach 900 S·cm^−1^ at 400 °C, meeting the requirements for the electrical conductivity of the cathode material samples [19].

**Figure 9:**
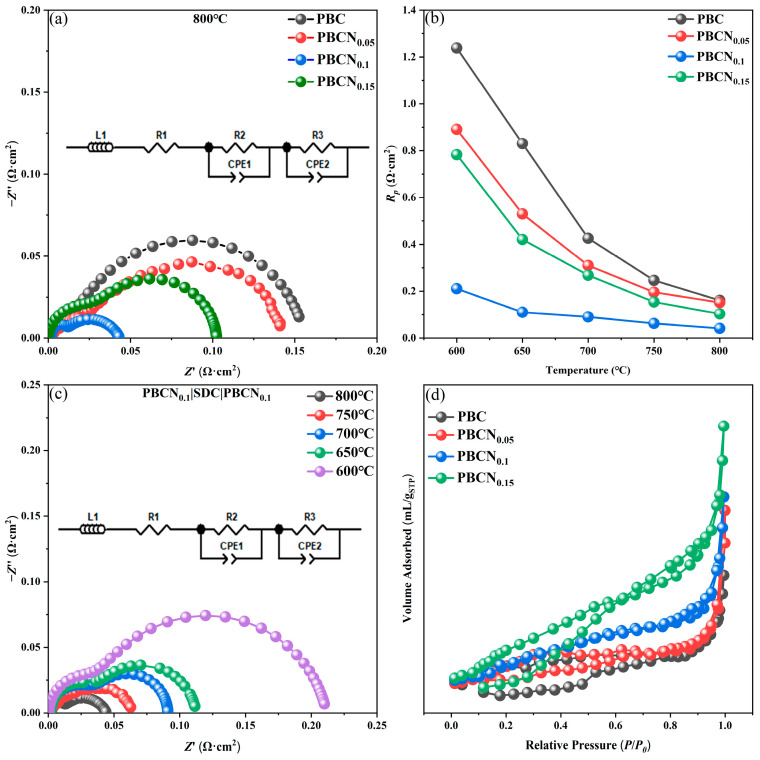
(**a**) Impedance diagram of different components at 800 °C. (**b**) Impedance line diagram of PBCNi_X_ series cathode material. (**c**) Impedance diagram of PBCNi_0.1_ at different temperatures. (**d**) BET diagram of PBCNi_X_.

Electrochemical Impedance Spectroscopy (EIS) is widely used in the field of electrochemistry as a key technique for characterizing the activity of electrochemical reactions to investigate the ORR reactivity of a material. The increase in power density was mainly related to the lower polarization impedance of the PBCNi_X_ cathode material. Ni doping improved the amount of Co in the cathode material, increased the oxygen diffusion coefficient, and improved the catalytic activity of ORR, resulting in a lower OCV of the material. The electrochemical characteristics of the PBCNi_X_ cathodes with a symmetrical cell structure and SDC electrolyte were investigated through AC impedance spectroscopy. Figure 9a shows that the polarization resistance (*R_p_*) of PBC at 800 °C was approximately 0.159 Ω·cm^2^, whereas the corresponding resistances of the PBCNi_X_ materials with X = 0.05, 0.10, and 0.15 were only approximately 0.146, 0.041, and 0.103 Ω·cm^2^, respectively. Ni ion doping remarkably reduced the polarization resistance at the cathode interface. As the Ni doping amount increased to 0.1, *R_p_* gradually decreased. Figure 9b provides the impedance spectra of the PBCNi_X_ series cathodes at 600–800 °C. After Ni was doped at the B site of PBCNi_X_, *R_p_* initially decreased and then increased. As illustrated in Figure 9c, when the Ni doping amount was 0.1, impedance continuously increased with the decrease in working temperature, reaching a minimum value of 0.041 Ω·cm^2^ at 800 °C. This finding indicated that Ni doping had a substantial effect on the electrochemical performance of the PBC cathode materials. The overall performance of SOFC cathodes has been found to depend mainly on O ion transport performance and the catalytic performance of ORR [20]. Excessive Co content can lead to a decrease in the oxygen vacancy coefficient δ. By doping Ni to improve the stoichiometry of Co in the cathode material, the oxygen vacancy coefficient can be increased and electrochemical impedance can be reduced. The performance of the PBC and PBCNi_X_ cathode materials depends not only on cathode conductivity but also on the catalytic activity of the cathode surface and gas transport rate through the porous cathode. Brunauer–Emmett–Teller (BET) pore size distribution tests were conducted to explore the catalytic activity of the cathode surface and gas transport rate through the porous cathode, and the results are provided in Figure 9d. With the doping of Ni ions, the specific surface area of the cathode materials gradually increased. This effect enhanced the exchange of the cathode with air oxygen, increased reaction activity, and reduced impedance. When the Ni doping amount was greater than 0.1, the polarization impedance showed an increasing trend again because with the continuous increase in the doping amount, the excessively high oxygen vacancy concentration caused defects in the cathode material and led to the localization of oxygen vacancies [21], thereby reducing the O ion transport rate and increasing *R_p_*. As shown in Table 3, the polarization impedance of PBCNi_0.1_ was lower than that of most Co-based materials. In summary, the PBCNi_0.1_ material can be considered a potential and promising cathode material with prospects for SOFCs.

**Figure 10:**
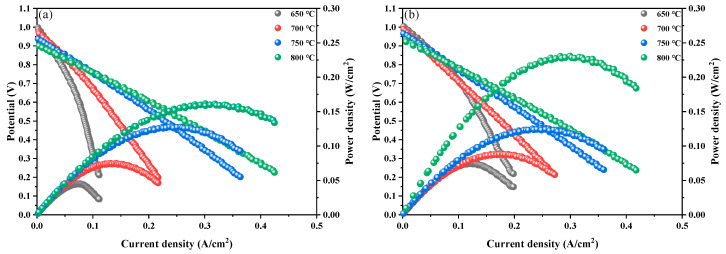
I-V-P curve of a single cell composed of (**a**) PBC and (**b**) PBCNi0.1 as a cathode at 650–800 °C.

An electrolyte-supported single cell has excellent performance in terms of heat resistance and mechanical strength because of its relatively hard electrolyte layer. This was chosen to construct and evaluate the output performance of PBCN_X_ as the cathode of SOFCs because of its simple preparation and the flexible selection of electrode materials. Figure 10a,b show the power densities of PBC and PBCN_0.1_ at 650–800 °C in a hydrogen atmosphere. The results indicate that the power density increased with the rise in temperature. At 650 °C, the open-circuit voltages (OCVs) were 0.99 and 1.0 V, which were lower than the theoretical voltage of 1.1 V [26]. These low OCVs were attributed to the partial reduction of Ce^4+^ in the SDC electrolyte into Ce^3+^ under the reducing atmosphere. This phenomenon led to electronic conductivity and internal short circuits [27]. At 800 °C, the power density of the single cell prepared with PBC was 161.1 mW·cm^−2^ and that of the single cell prepared with PBCNi_0.1_ increased by 69.5 mW·cm^−2^ to 230.6 mW·cm^−2^. This result indicated that Ni doping enhanced the output power density of the PBC cell and improved its catalytic activity.

## 3. Preparation and Characterization

### 3.1. Experimental Preparation

PrBaCo_1−X_Ni_X_O_3+δ_ (X = 0, 0.05, 0.1, 0.15) was prepared by using the sol–gel method with the reagents Pr(NO_3_)_3_·6H_2_O (AR, 99% Aladdin, WY, USA), Ba(NO_3_)_2_ (AR, 99.5% Aladdin, USA), Co(NO_3_)_2_·6H_2_O (AR, 99% Aladdin, USA), Ni(NO_3_)_2_·6H_2_O (AR, 98% Aladdin, USA), C_6_H_8_O_7_·H_2_O (AR, 99% Aladdin, USA), and C_10_H_16_N_2_O_8_ (AR, 99.5% Aladdin, USA). The raw materials were dissolved in deionized water as follows: metal ion: C_6_H_8_O_7_·H_2_O:C_10_H_16_N_2_O_8_ at a ratio of 1:1:1.5 [28]. The mixed solution was added to ammonia water and its pH was adjusted to 7–8. It was then placed in a water bath at a constant temperature of 80 °C and stirred until it formed a transparent purplish-red colloid. Subsequently, it was heated in a resistance furnace until the self-propagating reaction occurred. The prepared precursor was calcined at 1200 °C in a muffle furnace for 5 h at a heating rate of 3 °C /min to obtain the cathode powder PBCNi_X_. Each cathode powder was named PBCNi_X_ (X = 0, 0.05, 0.1, 0.15) in accordance with the different Ni doping amounts.

The electrolyte material Sm_0.8_Ce_0.2_O_2_(SDC) powder was sourced from Aladdin. The SDC electrolyte powder was pressed into a sheet with a diameter of 15 mm at 300 MPa. An SDC electrolyte sheet with a diameter of 15 mm and thickness of 0.6 mm was obtained after heating it at 1450 °C in a muffle furnace for 5 h.

The cathode material powder, terpinol, and ethyl cellulose were weighed in accordance with the mass ratio of 100:94:6 and then mixed and ground into a paste. The prepared cathode paste was uniformly coated on both sides of the electrolyte sheet through screen printing to form two symmetric cathodes. The resulting symmetric cell was then sintered in a high-temperature furnace to obtain the symmetric cell required for the electrochemical impedance (EIS) test [29]. The NiO–SDC composite anode was employed as the anode material of the cell (40% SDC electrolyte powder was added to the NiO powder). The NiO–SDC mixed powder and soluble starch (pore-creating agent) were placed in a ball mill tank in accordance with the mass ratio of 4:1, and an appropriate amount of alcohol was added for ball milling. A mixed slurry was obtained after 15 h of mixing. The required NiO–SDC composite anode powder was obtained after drying it.

### 3.2. Characterization

An X-ray diffractometer (Malvern Panalytical, Empyrean, Almelo, The Netherland) was used to analyze the crystal structure and phase composition of the synthesized samples with Cu Kα radiation (40 kV, 40 mA, and λ = 1.5418 Å) at a scan rate of 5°/min and scan range of 10–80°. The results were Rietveld-refined using the GSAS/EXPGUI software (PC-GSAS 1). The elemental distribution and lattice spacing of the material were also characterized using transmission electron microscopy (TEM, JEOL, 2100F, Tokyo, Japan). X-ray photoelectron spectroscopy (XPS, Thermofisher Scienticfic, ESCALAB250XI, Waltham, MA, USA) was employed to analyze the valence state of each element in the materials. A high-resolution transmission electron microscope (HR-TEM, FEI, TecnaiF20, Hillsboro, OR, USA) was utilized to analyze the diffraction fringe width of the materials equipped with an energy dispersive spectrometer (EDS) employed to detect the distribution of each element. The cross-section morphology of the symmetric cells was investigated with a scanning electron microscope (TESCAN, CAIA3, Brno, Czechia). The TEC of the cathode materials was tested with a thermal dilatometer (NETZSCH, DIL402C, Selb, Germany). The test atmosphere was high-purity air. The temperature range was 30–750 °C, and the heating rate was 5 °C/min. Electronic paramagnetic resonance (EPR) tests were performed at room temperature by the Electron Paramagnetic Resonance Spectrometer (Bruker, EMX PLUS, Saarbryucken, Germany). Specific surface area and pore size analyses were performed by a physical adsorption instrument (ASAP 2460, micromeritics, Houston, TX, USA).

### 3.3. Electrochemical Test

**Figure 11:**
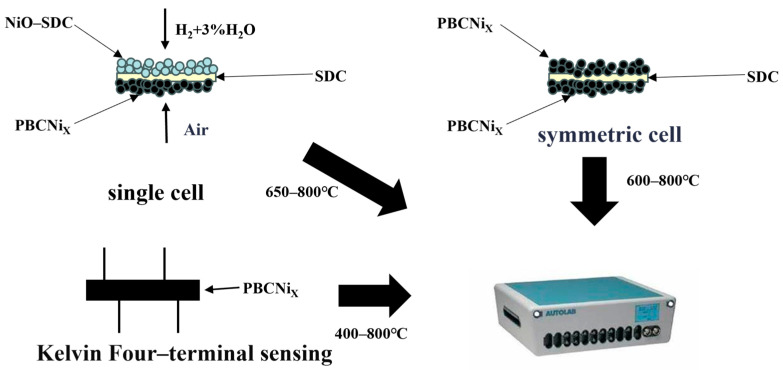
Diagram of electrochemical performance test.

A PGSTAT302N-type electrochemical workstation (Autolab, PGSTAT302N, Chișinău, Moldova) was used to test the conductivity at 200–800 °C and the electrochemical impedance spectrum of the symmetrical cell was obtained. The two voltage ends in the middle of the strip sample were connected to the induction and reference electrodes of the workstation and the two outer current sections were connected to the working and auxiliary electrodes of the workstation. The sample was placed in a tube furnace equipped with an electrochemical workstation to test the conductivity of the strip samples. The test temperature range was 400–800 °C with an interval of 50 °C. The conductivity of the samples at different temperatures was measured. The symmetrical cell was prepared for EIS. The test temperature range was 600–800 °C with an interval of 50 °C in an air atmosphere. The test frequency range was 100 kHz–0.1 Hz and the amplitude was 10 mV. The test was conducted in RMS mode. The output power of a single cell was tested by using SDC as an electrolyte and NIO–SDC as an anode. The anode side was fed with wet hydrogen (H_2_ + 3% H_2_O) as a fuel gas at a rate of 30 mL/min and the cathode side was directly in contact with air. The output power density of the single cell was tested with a range of 600–800 °C and an interval of 50 °C. The instruments, materials, and conditions used for electrochemical performance testing are shown in Figure 11.

## 4. Conclusions

The experimental results show that the TEC and polarization resistance of PBCNi_X_ decreased significantly with the increase in Ni substitution and the oxygen vacancy concentration and ORR activity increased. TEC decreased with the increase in Ni. When the doped amount of Ni was 0.15, the TEC was 18.0548 × 10^−6^ K^−1^, which was a low value for Co-based cathode materials. Although Ni doping could effectively reduce the polarization resistance of the Pr_0.5_Ba_0.5_Co_1−X_Ni_X_O_3+δ_ cathode materials, excessive doping had adverse effects, and the doping amount of 0.1 had the best effect among all the tested doping amounts. At 800 °C, the polarization resistance of the Pr_0.5_Ba_0.5_Co_0.9_Ni_0.1_O_3+δ_ cathode material was 0.041 Ω·cm^2^. The output power density of Pr_0.5_Ba_0.5_Co_0.9_Ni_0.1_O_3+δ_ increased by 69.5 mW·cm^−2^ at 800 °C. Therefore, doping of the Ni element effectively reduced the TEC and polarization impedance of the material, increasing the service life of the cell. The material is expected to become an alternative cathode material with broad development prospects.

## Figures and Tables

**Table 1:** Refined Rietveld data of PBCNi_X_ samples.

Sample	Space Group	*a* (Å)	*c* (Å)	*V* (Å)	*x* ^2^	*R_wp_* (%)	*R_p_* (%)
PBC	P4/mmm	3.903786	7.627325	116.237	2.02	9.68	7.77
PBCN_0.05_	P4/mmm	3.909524	7.636684	116.722	2.39	8.93	7.91
PBCN_0.1_	P4/mmm	3.912615	7.642894	117.002	2.45	9.87	8.30
PBCN_0.15_	P4/mmm	3.914977	7.646315	117.195	2.50	9.47	8.09

**Table 2:** TEM-EDS results of PBC and PBCNi_0.1_ cathode materials.

Element	PBC Atom Ratio (%)	PBCNi_0.1_ Atom Ratio (%)
O	61.37	61.29
Co	19.79	17.68
Ni	-	1.83
Ba	9.67	9.81
Pr	9.17	9.39
Total	100.00	100.00

**Table 3:** The polarization impedance *R_p_* of PBCNi_X_ cathode material and other Co-based materials.

Sample	Electrolyte	Temperature (°C)	*R_p_* (Ω·cm^2^)	TEC (K^−1^) (30–800 °C)	Reference
PBCF	LSGM	700	0.221	21.0 × 10^−6^	[22]
PBC	LSGM	700	0.07	23.5 × 10^−6^	[23]
LBSC	SDC	800	0.081	26.2 × 10^−6^	[24]
NBC	LSGM	800	0.078	17.1 × 10^−6^	[25]
PBCNi_0.1_	SDC	700	0.09	19.4837 × 10^−6^	This work
PBCNi_0.1_	SDC	800	0.041	19.4837 × 10^−6^	This work

## Data Availability

The original contributions presented in this study are included in the article/Supplementary Material. Further inquiries can be directed to the corresponding author(s).

## Supplementary Materials

The following supporting information can be downloaded at: https://www.mdpi.com/article/10.3390/molecules30071482/s1, Figure S1: EPR signal intensity map of oxygen vacancies in PBCNi_X_ cathode material; Table S1: O1s peak-differentiating fitting results of PBCNi_X_ cathod materials; Table S2: Co2p peak-differentiating fitting results of PBCNi_X_ cathod materials (1); Table S3: Co2p peak-differentiating fitting results of PBCNi_X_ cathod materials (2).
