# Supplementary material for: Ni-Doped Pr0.5Ba0.5CoO3+δ Perovskite with Low Polarization Resistance and Thermal Expansivity as a Cathode Material for Solid Oxide Fuel Cells
Source: Molecules. 2025 Mar 27;30(7):1482. doi: 10.3390/molecules30071482 (PMC11990097; doi:10.3390/molecules30071482)
Supplement: Supplementary file 1 [file molecules-30-01482-s001.zip › molecules-3467069-supplementary.pdf]

Table S1 O1s peak-differentiating fitting results of $PBCNi_x$ cathod materials

Sample	$O_{adsorbed}$ (eV)	FWHM (eV)	$O_{adsorbed}$ (%)	$O_{lattice}$ (eV)	FWHM (eV)	$O_{lattice}$ (%)	$O_{vacancy}$ (eV)	FWHM (eV)	$O_{vacancy}$ (%)
PBC	531.52	2.41	75.10	528.37	0.97	16.02	529.07	1.45	8.88
$PBCN_{0.05}$	531.45	2.49	76.22	528.31	1.07	14.25	529.05	1.41	9.53
$PBCN_{0.1}$	531.37	2.32	76.31	528.40	0.97	12.68	529.11	1.34	11.01
$PBCN_{0.15}$	531.34	2.23	76.74	528.28	1.01	11.90	528.98	1.34	11.36

Table S2 Co2p peak-differentiating fitting results of $PBCNi_x$ cathod materials (1)

Sample	Co^{3+} 2p1/2 (eV)	Co^{2+} 2p1/2 (eV)	Co^{3+} 2p3/2 (eV)	Co^{2+} 2p3/2 (eV)	Co^{3+} (%)	Co^{2+} (%)
PBC	793.46	795.71	778.12	780.38	37.19	62.81
$PBCN_{0.05}$	793.49	795.60	778.12	780.25	40.79	59.21
$PBCN_{0.1}$	793.55	795.54	778.31	780.15	42.12	57.89
$PBCN_{0.15}$	793.41	795.50	778.14	780.23	42.56	57.44

Table S3 Co2p peak-differentiating fitting results of $PBCNi_x$ cathod materials (2)

Sample	Co^{3+} 2p1/2 (eV)	Co^{3+} 2p1/2 FWHM (eV)	Co^{3+} 2p1/2 CPS.eV	Co^{3+} 2p3/2 (eV)	Co^{3+} 2p3/2 FWHM (eV)	Co^{3+} 2p3/2 CPS.eV
PBC	793.46	1.68	28398.91	778.12	1.42	42295.95
$PBCN_{0.05}$	793.49	1.67	29700.51	778.12	1.33	38170.55
$PBCN_{0.1}$	793.55	2.02	13623.13	778.31	1.89	15932.05
$PBCN_{0.15}$	793.41	1.67	11271.8	778.14	1.96	18998.0
Sample	Co^{2+} 2p1/2 (eV)	Co^{2+} 2p1/2 FWHM (eV)	Co^{2+} 2p1/2 CPS.eV	Co^{2+} 2p3/2 (eV)	Co^{2+} 2p3/2 FWHM (eV)	Co^{2+} 2p3/2 CPS.eV
PBC	795.71	2.24	41816.88	780.38	2.33	77492.08
$PBCN_{0.05}$	795.60	2.08	33046.34	780.25	2.33	65422.38
$PBCN_{0.1}$	795.54	1.86	14269.27	780.15	1.83	25616.88
$PBCN_{0.15}$	795.50	2.02	13901.8	780.23	1.99	27662.83

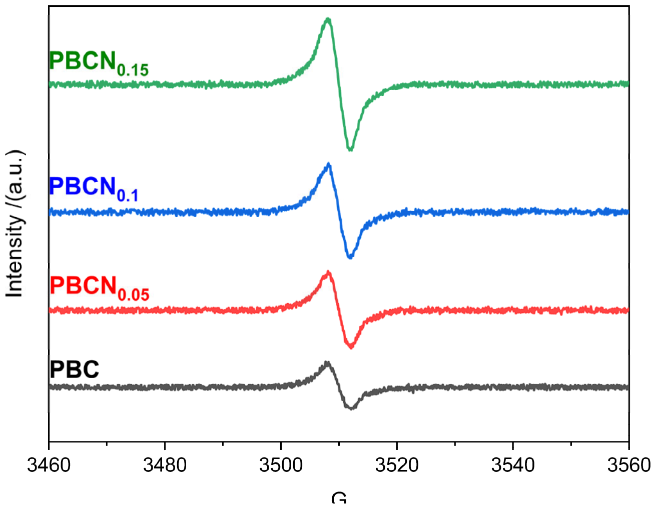

Figure S1 EPR signal intensity map of oxygen vacancies in PBCNix cathode material.
